# Molecular markers associated with aluminium tolerance in *Sorghum bicolor*

**DOI:** 10.1186/s41065-018-0059-3

**Published:** 2018-04-13

**Authors:** Emily Jepkosgei Too, Augustino Osoro Onkware, Beatrice Ang’iyo Were, Samuel Gudu, Anders Carlsson, Mulatu Geleta

**Affiliations:** 1 Department of Biological Sciences, University of Eldoret, P.O. Box 1125-30100, Eldoret, Kenya; 2 Rongo University College, P.O. Box 103-40404, Rongo, Kenya; 3 Department of Plant Breeding, Swedish University of Agricultural Sciences, P.O. Box 101, SE-230 53 Alnarp, Sweden

**Keywords:** Aluminium tolerance, Mapping population, Molecular markers, Net root length in aluminium, *Sorghum bicolor*

## Abstract

**Background:**

Sorghum (*Sorghum bicolor*, L. Moench) production in many agro-ecologies is constrained by a variety of stresses, including high levels of aluminium (Al) commonly found in acid soils. Therefore, for such soils, growing Al tolerant cultivars is imperative for high productivity.

**Methods:**

In this study, molecular markers associated with Al tolerance were identified using a mapping population developed by crossing two contrasting genotypes for this trait.

**Results:**

Four SSR (*Xtxp34*, *Sb5_236*, *Sb6_34*, and *Sb6_342*), one STS (*CTG29_3b*) and three ISSR (*811_1400*, *835_200* and *884_200*) markers produced alleles that showed significant association with Al tolerance. *CTG29_3b, 811_1400*, *Xtxp34* and *Sb5_*236 are located on chromosome 3 with the first two markers located close to *Alt*_*SB*_, a locus that underlie the Al tolerance gene (*SbMATE*) implying that their association with Al tolerance is due to their linkage to this gene. Although *CTG29_3b* and *811_*1400 are located closer to *Alt*_*SB*_, *Xtxp34* and *Sb5_236* explained higher phenotypic variance of Al tolerance indices. Markers *835_200*, *884_200*, *Sb6_34* and *Sb6_342* are located on different chromosomes, which implies the presence of several genes involved in Al tolerance in addition to S*bMATE* in sorghum.

**Conclusion:**

These molecular markers have a high potential for use in breeding for Al tolerance in sorghum.

## Background

Sorghum (*Sorghum bicolor* L. Moench), is a staple cereal in many parts of Africa and Asia where it is grown mostly on small-scale, resource poor holdings. Although it is a hardy cereal, its production is significantly reduced by aluminium (Al) stress, especially in the highly leached acidic soils [1, 2]. Inhibition of root growth is the primary symptom of Al stress in plants [3, 4]. The primary consequence of Al stress is a poorly developed root system that makes the crop vulnerable to drought and limited nutrient uptake [5] and ultimately reduced crop yields [6]. In Kenya, over 70% of sorghum is produced in the western and central regions. These areas are characterized by acid soils [7] with Al percent saturation ranging from 4 to 46% [8]. Low sorghum grain yield in the country is partly attributed to acid soil stress [9] including Al toxicity. Therefore, there is the need to deploy Al tolerant sorghum cultivars in order to increase productivity.

Plant tolerance to aluminium stress is based on exclusion or internal mechanisms that enable Al to be tolerated once it has entered the plant cells [10, 11]. Some plant species accumulate Al and complexed it with other substances to render it less toxic [12]. Al tolerant cultivars of sorghum and most other cereals use the Al exclusion mechanisms based mainly on secretion of organic acids, such as citrate, malate and oxalate that chelate Al outside the cells and thereby reduce its availability [13, 14]. When exposed to Al stress, tolerant sorghum varieties reportedly secrete large quantities of citric acid [14], malic acid and trans-aconitic acids [15, 16]. Aluminium-induced exudation of organic acids in plant roots is mediated by anionic channels in the plasma membrane [12]. The plant genes involved in the Al-induced acid exudation are members of the aluminium activated malate transporter (ALMT) and the multi-drug and toxin extrusion (MATE) families that encode membrane transporter proteins [14, 17, 18].

Although significant progress has been made in crop improvement through phenotypic selection for Al tolerance [5, 19–21], the testing procedures may be difficult and time consuming due to the effect of genotype by environment interactions for this trait. In this regard, molecular markers based screening procedure can be more efficient than the use of morphological markers in identifying Al tolerant sorghum genotypes.

Sorghum has significant genotypic variation for tolerance to Al [22, 23], which can be exploited to breed genotypes with superior tolerance to Al stress. Significant progress has been made in developing genomic tools and resources related to aluminium tolerance in sorghum, including the development of some molecular markers that can be used for marker assisted selection [14, 23–27]. However, additional work has to be done through exploring global sorghum genetic resources to identify additional genomic regions that contribute to this trait and eventually develop sets of molecular markers that can be used to efficiently breed sorghum for Al tolerance.

An aluminium tolerance locus referred to as *Alt*_*SB*_ was identified through comparative mapping procedure in sorghum [24]. This locus was later found to underlie *SbMATE,* a MATE family gene, which encodes a protein that is responsible for citric acid exudation [14]. Causative polymorphisms that include insertions and single nucleotide polymorphism (SNPs) that were positively correlated with aluminium tolerance have been identified within this gene [27]. STS markers designated as *CTG29* and *M181* have been reported to be closely linked to *Alt*_*SB*_ at 0.2 cM and 0 cM, respectively [14]. However, it was not known whether the sorghum population used in this study relied on *Alt*_*SB*_ or different Al tolerance gene(s). Conserved gene order in genomic regions harboring Al tolerance loci has been reported in members of the grass family [23]. Hence, with the availability of saturated genetic maps of major cereals, it is possible to identify markers linked to Al tolerance genes in sorghum that are orthologous to Al tolerance genes in other cereal crops. The present study was conducted to identify molecular markers associated with genes/QTLs that confer tolerance to aluminium toxicity in sorghum.

## Methods

### Plant material and the development of the mapping population

Two sorghum lines, *Seredo* and *ICSR 110,* were used to develop a mapping population for the identification of molecular markers associated with aluminium tolerance. *Seredo* is a popular but Al-sensitive commercial Kenyan sorghum with medium height and early maturity that is marketed by the Kenya Seed Company (MUSRT). *ICSR 110* is an early maturing, medium height, Al-tolerant inbred line developed by International Crops Research Institute for the Semi-Arid Tropics (ICRISAT, Hyderabad, India). *Seredo* was used as a pollen recipient from *ICSR 110* to develop the hybrid population that was advanced to F_3_ by selfing. The mapping population comprised 229 F_2:3_ progenies derived from 22 F_2_ plants, which in turn derived from three different F_1_ plants.

The Al tolerance of parental lines and F_2:3_ progenies was evaluated according to the procedures described by Magalhaes et al. [24] using the basal nutrient solution of Magnavaca et al. [28]. A concentration of 148 μM Al was used to study the effect of aluminium on root growth of the seedlings based on recommendations from previous studies [14, 23]. For this purpose, seeds were sterilized in 1% hypochlorite and germinated on paper.

Since individual plants in F_2:3_ population are not genetically identical, it was not possible to set up a separate control experiment. Hence, the following procedure was followed to determine the root growth of individual plants under a solution without Al (control solution) and under a solution containing Al, as described in Caniato et al. [23] and Ringo et al. [29]. The two solutions have the same composition except that the latter contains 148 μM Al. First, the seedlings were given a germination period of 4 days and the root length of each plant, which is referred to as initial root length under control solution (il_c_), was measured. Then, the seedlings were acclimated in a control nutrient solution for 24 h (1 day) and the root length of each plant, which is referred to as final root length under control solution (fl_c_), was measured. The seedlings were then transferred to a solution containing 148 μM Al and allowed to grow for 5 days and the root length of each plant, which is referred to as final root length under Al (fl_Al_), was measured. Based on these measurements, the net root length of each plant under Al (NRL_Al_) was calculated as fl_Al_-fl_c_, whereas the percent relative root growth of each plant under the Al solution as compared to the growth under the control solution (%RRG) was calculated as [(fl_Al_–fl_c_)_5d_/(fl_c_–il_c_)_1d_ × 5] × 100. The values of NRL_Al_ and %RRG were used to classify the F_2:3_ progenies into tolerant or sensitive groups. Leaves were sampled from the parents, F_1_ and F_2:3_ seedlings for DNA extraction and marker analysis.

### DNA extraction

Genomic DNA was extracted separately from leaf tissue of each F_2:3_ seedling, parent and F_1_ hybrid using a cetyltrimethyl ammonium bromide (CTAB) method, as described in Bekele et al. [30]. In total, DNA was extracted from six parental plants (three *ICSR 110* and three *Seredo*), three F_1_ and 229 F_2:3_ plants. The DNA quality and concentration were assessed using a Nanodrop® ND-1000 spectrophotometer (Saveen & Werner®, Malmö, Sweden) and by ethidium bromide staining following electrophoresis on 1.5% agarose gel.

### PCR amplification of ISSR, SSR and STS markers

Fifty (50) inter-simple sequence repeat (ISSR) primers, twenty four (24) simple sequence repeat (SSR) primer-pairs and two (2) sequence tagged site (STS) primer-pairs were tested on Al tolerant and sensitive parental lines to identify potential markers linked to Al tolerance.

The 50 ISSR primers were selected for screening, as they generated clear and well-separated banding pattern suitable for detecting polymorphism. The ISSR amplification reaction was performed in a total volume of 25 μl containing 1× reaction buffer (20 mM Tris-HCl pH 8.55, 16 mM (NH_4_)SO_4_, 0.01% Tween®20 and 2 mM MgCl_2_), 0.4 μM primer, 0.2 mM dNTPs, 0.5 U *Taq* DNA polymerase and 10 ng of sample DNA. Amplifications were carried out using GeneAmp® PCR System 9700 (Applied Biosystems) with an initial denaturation step at 94 °C for 1 min followed by 40 cycles of 94 °C for 1 min, 55 °C for 2 min and 72 °C for 18 s; and a final extension step at 72 °C for 5 min.

The 24 SSR markers were selected based on their representation of all sorghum chromosomes and their previously reported high polymorphism. The PCR amplification of SSR loci was performed in a 25 μl reaction mixture containing 25 ng of template DNA, 1× PCR buffer [20 mM Tris-HCl (pH 8.55), 16 mM (NH_4_)_2_SO_4_, 0.01% Tween 20 and 2 mM MgCl_2_], 0.3 mM dNTPs, 0.25 μM of each primer, and 1 U Taq DNA polymerase (Saveen & Werner®, Sweden). The PCR reactions employed a touchdown PCR method [31] and were run using an Eppendorf® AG-22331 Thermal Cycler (Hamburg, Germany) after optimizing annealing temperatures for the individual primer-pairs. The amplification profiles consisted of initial denaturation of the template DNA at 95 °C for 3 min, followed by 10 cycles at 95 °C for 30 s, 60 °C or 65 °C (depending on the primer-pair) for 30 s (with a decrease of 1 °C/cycle) and 72 °C for 45 s; 30 cycles at 94 °C for 30 s, 50 °C or 55 °C (depending on the primer-pair) for 30 s and 72 °C for 45 s, and a final extension for 20 min at 72 °C.

The two STS markers (*CTG29* and *M181*) are tightly linked to *Alt*_*SB*_, the locus that underlies *SbMATE* Al tolerance gene [14, 23]. The primers used for the amplification of these markers were those published by Caniato et al. [23]. The PCR reactions for STS markers were run in a GeneAmp® PCR System 9700 Thermal Cycler (Applied Biosystems) in a 20 μl reaction mixture containing 30 ng of DNA template, 1× PCR buffer [20 mM Tris-HCl (pH 8.55), 16 mM (NH_4_)_2_SO_4_, 0.01% Tween 20 and 2 mM MgCl_2_], 0.5 mM dNTPs, 1.9 mM MgCl_2_, 0.11 μM of each primer and 1 U Taq DNA polymerase. The PCR program consisted of an initial DNA denaturation step of 1 min at 95 °C followed by 30 cycles of denaturation at 95 °C for 30 s, annealing at 55 °C (for *M181*) or 57 °C (for *CTG29*) for 1 min, extension at 72 °C for 1 min, followed by a final extension step at 72 °C for 5 min. In the case of *CTG29*, additional 2 mM MgCl_2_ was used in the reaction mixture, and 40 cycles of amplification at annealing temperature of 58 °C was used.

### Electrophoresis, staining, visualization and polymorphism survey

The PCR products were separated on 1.5% agarose gel containing ethidium bromide to confirm amplification and thereafter visualized and photographed using a UV photo print system (IP-215-SD) fitted with a Sony® XC-ST50CE camera (Saveen & Werner, Sweden). For both ISSR and SSR markers, the PCR products were then electrophoresed on polyacrylamide gels (CleanGel 10% 52S; ETC Electrophorase-technik®, Germany) for better resolution and silver-stained, as described by Geleta and Bryngelsson [32].

A marker polymorphism survey of the parents was done and the markers that differentiated the two parents were used to genotype the F2:3 progenies. The F_1_s were included in the analysis as positive controls. Three ISSR primers, four SSR and one STS primer-pairs generated promising markers (Table 1). Two of the three selected polymorphic ISSR markers were converted to sequence characterized amplified region (SCAR) markers as described below.

**Table 1:** List of primers/primer-pairs used to amplify markers that showed association with Al tolerance in sorghum

Marker type	Locus name	Primer sequences (5′ - 3′)	Repeat motif	Fragment size range (bp)	T_a_ (°C)
ISSR	*ISSR_811* ^a^	F/R: GAG AGA GAG AGA GAG AC	–	1400	55
	*ISSR_835* ^a^	F/R: AGA GAG AGA GAG AGA GCT C	–	Ca. 200	55
	*ISSR_884* ^a,i^	F/R: HBH ATC AGA GAG AGA GAG AG	–	Ca. 200	55
SSR	*Sb5_236* ^b^	F: GCC AAG AGA AAC ACA AAC AA R: AGC AAT GTA TTT AGG CAA CAC A	(AG)_20_	160–208	55
	*Sb6_342* ^b^	F: TGC TTG TGA GAG TGC CTC CCT R: GTG AAC CTG CTG CTT TAG TCG ATG	(AC)_25_	270–294	50
	*Sb6_34* ^b^	F: AAC AGC AGT AAT GCC ACA C R: TGA CTT GGT AGA GAA CTT GTC TTC	[(AC)/(CG)]_15_	188–208	55
	*Xtxp34* ^c^	F: TGG TTC GTA TCC TTC TCT ACA G R: CAT ATA CCT CCT CGT CGC TC	(CT)_29_	365	55
STS	*CTG29* ^d,e^	F: ATG CAG TAT CTG CAG TAT CAT TT R: AAT CCG TCA GGT CAG CAA TC	–	226–228	57
	*M181* ^d,e^	F: AAG GCA ACA ACT GAG GCA CT R: TCG CTA GAG TGG TGC AAG AA	–	169–174	55
	*CTG29_3b* ^f,g^	F: TGG TGA TAT TAT TAA AAC TGT GTT A R: AAT CCG TCA GGT CAG CAA TC	–	200	58
SCAR	*SCAR_811* ^h^	F: ACG CAA GTT CCG AGG AGA A R: GAG AGA GAG AGA GAG ACA GAG GTT GTC	–	1119	65
	*SCAR_884* ^h^	F: AGA GAG AGA GAG AGC TCA CAC A R: AGA GAG AGA GAG AGG TGT TTT A	–	226 and 446	61

^a^Source: University of British Columbia, Canada: http://www.scribd.com/doc/23812434/UBC

^b^Source: Smith et al. [54], ^c^Source: Kong et al. [55], ^d^Source: Caniato et al. [23]; ^e^The markers did not differentiate the resistant and sensitive parents in this study. ^f^Source of forward primer: this study; ^g^Source of reverse primer: Caniato et al. [23]; ^h^Source: this study. F = Forward; R = Reverse; F/R = used as both forward and reverse primers. ^i^In the primer sequence of *ISSR_884*, B = C or G or T whereas H = A or C or T

### Development and analysis of SCAR markers

Two DNA fragments of approximately 1400 bp and 200 bp long that were amplified by *ISSR_811* and *ISSR_884* primers, respectively, were identified as co-segregating with aluminium tolerance. In this paper, these fragments are referred to as *811_1400* and *884_200*, respectively. Both fragments were recovered from gels for cloning and sequencing. The *811_1400* fragment was purified from agarose gel using QIAquick®Gel extraction and purification kit (QIAGEN GmbH, Germany) according to the manufacturer’s instructions. The purified DNA was used in the subsequent cloning of the marker fragment.

The *884_200* fragment was not clearly resolved on agarose gel and hence the PCR products were separated on polyacrylamide gel and then recovered following the procedure described by Sanguinetti [33]. The fragments were extracted from agarose or polyacrylamide gels in 100 μl of extraction buffer (10 mM Tris-HCl, 50 mM KCl, 1.5 mM MgCl_2_, 0.1% Triton® X-100, pH 9.0) by incubation at 95 °C for 20 min. One microliter of the extracted DNA solution was used to re-amplify the target fragment using Advantage® HF2 Taq polymerase (Invitrogen) according to the ISSR amplification procedures describe above. An aliquot of the PCR products was analysed on agarose gel to confirm amplification and the rest purified using QIAquick® PCR purification kit (QIAGEN GmbH, Germany). The purified DNA fragments were ligated into pJET1.2/blunt cloning vector following the sticky-end cloning protocol as outlined in the CloneJET^™^ PCR Cloning Kit instruction manual (Fermentas®, Life Sciences).

Transformation of chemically competent *Escherichia coli* cells (One Shot® TOP10-Invitrogen) was done following the manufacturer’s instructions. Single colonies of transformed cells were picked and analyzed by colony PCR using pJET1.2 primer-pairs to identify clones that carried the fragments of interest. These clones were sub-cultured in liquid Luria Bertini (LB) media containing ampicillin (100 μg/ml) for plasmid mini-preparations. Plasmid DNA was purified using QIAprep® spin miniprep kit (QIAGEN GmbH, Germany) as described by the manufacturer.

Plasmid DNA harbouring the inserts was digested with *Bgl*11 restriction endonuclease to confirm fragment size. Samples that had the desired inserts were sequenced using one of the pJET1.2 primers. The sequence quality was checked using Sequence Scanner v. 1.0 (Applied Biosystems®) and sequence alignment was done with ClustalX v. 2 [34]. The aligned sequences were manually edited using BioEdit© version 7.0 [35].

The sequences of the *811_1400* and *884_200* fragments were used to design new primers to amplify SCAR markers *SCAR_811* and *SCAR_884*, respectively. Primers were designed by extending the ISSR primers so that the 3′-end of the primers contain SNPs that differentiated the tolerant and sensitive sorghum genotypes. Primer3 (an online primer designing program (http://primer3.ut.ee) was also used to design alternative primers. Different combinations of primers were tested for optimal amplification of the corresponding SCAR markers.

The PCR for *SCAR_884* was performed in a volume of 50 μl containing 50 ng of template DNA, 1× PCR buffer, 3.5 mM MgCl_2_, 0.1 mM dNTPs, 0.5 μM of each primer and 1 U of Taq DNA polymerase. The reactions were carried out in GeneAmp® PCR System 9700 Thermal Cycler with an initial denaturation step at 94 °C for 3 min, followed by 30 cycles at 94 °C for 1 min, 61 °C for 1 min, and 72 °C for 2 min, followed by final extension step at 72 °C for 7 min. The reaction mixtures and PCR conditions for *SCAR_811* were similar to those of *SCAR_884* except that the concentration of MgCl_2_ was 2 mM and the annealing temperature was 65 °C.

### Data scoring and statistical analysis

The bands for ISSR, STS and SCAR markers were scored as present (1) or absent (0) whereas the SSR marker fragments were scored as either parental allele (A or B),or heterozygous for both alleles (AB). All phenotypic data were subjected to analysis of variance (ANOVA) using SPSS® and means were separated using Tukey’s test at 5% significance level. The association of individual marker with Al tolerance was tested with one-way analysis of variance and linear regression analysis, with a threshold significance level of *P* ≤ 0.05. When a significant difference was found between the genotypic groups, a pairwise comparison was made using Tukey’s test. For those markers that showed significant association with aluminium tolerance when analyzed individually, multiple linear regression analysis was conducted to check for collinearity between the markers. The Chi-square goodness-of-fit test for the Mendelian segregation of the alleles of each marker was also conducted for the F_2:3_ population (Table 2).

**Table 2:** Simple and multiple linear regression analysis of 229 F_2:3_ progeny plants for the association between marker-based genotypes and net root length in aluminium (NRL_Al_) and percent relative root growth (%RRG); and Chi-square goodness- of-fit test for Mendelian segregation of the markers

Marker	^d^Chromosome number	^d^Marker position (bp range)	NRL_Al_			%RRG			χ ^2^ test	
			F	*P*-value	R^2^	F	*P*-value	R^2^	χ^2^	*P*-value
*Sb5_236*	3	52,278,272–52,278,445	15.08	0.000	0.110	11.92	0.001	0.050	5.33	0.070
*Xtxp3*4	3	69,704,047–69,704,411	18.75	0.000	0.142	17.07	0.000	0.070	3.94	0.139
*CTG29_3b*	3	70,939,651–70,939,834	11.42	0.001	0.048	9.49	0.002	0.040	3.60	0.059
*811_1400* ^a^	3	71,473,923–71,475,318	24.88	0.000	0.100	9.78	0.002	0.041	0.14	0.709
*884_200* ^b^	6	42,689,435–42,689,555	4.64	0.032	0.017	5.07	0.025	0.025	3.09	0.079
*Sb6_342*	7	38,823,193–38,823,478	4.49	0.012	0.038	6.06	0.015	0.030	0.36	0.837
*Sb6_34*	8	61,802,592–61,802,795	7.58	0.001	0.063	4.02	0.046	0.020	5.70	0.058
*835_200*	10	56,491,522–56,491,702	3.61	0.049	0.016	6.85	0.009	0.029	1.50	0.220
Four markers^c^	3		15.67	0.000	0.219	7.06	0.000	0.119		
All markers			8.15	0.000	0.251	3.82	0.000	0.122		

^a^Ccorresponds to *SCAR_811;* ^b^corresponds to *SCAR_884*; ^c^the four markers on chromosome 3. ^d^The chromosome number and the position of the markers within the corresponding chromosomes (in bp range) were identified through BLAST (https://blast.ncbi.nlm.nih.gov/Blast.cgi) search of the sequences of the markers against the sorghum genome. The GenBank Accession number of chromosomes 3, 6, 7, 8 and 10 are *NC_012872.2*, *NC_012875.*2, *NC_012876.*2, *NC_012877.2*, *NC_012879.2*, respectively. Note: the position of *SbMATE* gene on chromosome 3 (*NC_012872.2*) is 71,105,461–71,108,054 bp

## Results

### Response of parental lines and their progenies to aluminium stress

**Fig. 1:**
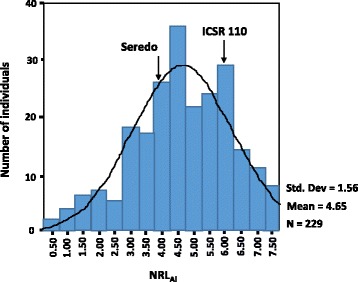
Frequency distribution of NRL_Al_ of 229 F_2:3_ progeny derived from a cross between *Seredo*, an aluminium sensitive sorghum line, and *ICSR 110*, an aluminium tolerant line. The NRL_Al_ mean values for *Seredo* and *ICSR 110* were 3.8 (*n* = 40) and 6.0 (*n* = 40), respectively as shown by the arrows

**Fig. 2:**
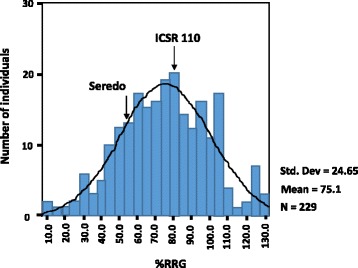
Frequency distribution of %RRG of 229 F_2:3_ progeny derived from a cross between *Seredo,* an aluminium sensitive sorghum line, and *ICSR 110,* an aluminium tolerant line. The %RRGs of the parental lines are shown by the arrows for comparison

The root growth in the two parental sorghum lines, *Seredo* and *ICSR 110,* exhibited significantly different responses (*p* < 0.05) to the Al stress at 148 μM Al (Figs. 1 and 2). *ICSR 110* was tolerant with only 15% reduction in root growth, whereas *Seredo* had 53% reduction in root growth. The net root growth in aluminium and percentage of relative root growth were strongly correlated (*r*^2^ = 0.70). Based on the variation in net growth of the sensitive parent, the F_2:3_ progeny plants with net root growth of 4.0 cm or less were classified as sensitive. Those that had net root growth ranging between 4.0 cm and 5.0 cm were classified as moderately tolerant, while progeny plants with a net root growth above 5.0 cm were classified as tolerant to Al stress. Based on this criterion, among the 229 F_2:3_ population, 98, 58 and 73 individuals were tolerant, moderately tolerant and sensitive, respectively.

### The molecular markers associated with tolerance to Aluminium toxicity

Ten of the fifty ISSR primers amplified fragments that were polymorphic between the two parental lines. Preliminary analysis of the ten polymorphic markers using 21 Al tolerant and 21 Al sensitive F_2:3_ progeny revealed that *ISSR_811*, *ISSR_835* and *ISSR_884* primers amplified three fragments of approximately 1400 bp, 200 bp and 200 bp, respectively, having uneven distribution among aluminium tolerant and sensitive progenies. After genotyping, the remaining 187 F_2:3_ progeny plants were genotyped with these primers. The analysis of phenotypic and genotypic data from the 229 F_2:3_ progeny revealed significant association of these markers with tolerance to Al toxicity (Table 2). The *811_1400* fragment was specific to *Seredo* and found in most Al sensitive progeny. On the other hand, the *835_200* and *884_200* amplified fragments showed significant association with aluminium tolerance.

Basic Local Alignment Search Tool (BLAST) was used to search the GenBank (https://www.ncbi.nlm.nih.gov/genbank/) and the Gramene (http://www.gramene.org) databases to locate the genomic positions of these markers within the sorghum genome. The BLAST searching of *811_1400* marker sequence in the Gramene database resulted in a sorghum sequence that share 1386 bp (with 99.6% sequence identity) with *811_1400* on chromosome 3 at position 71,512,250–71,513,635 bp. The corresponding position of this marker on sorghum chromosome 3 (GenBank accession number *NC_012872.2*) is 71,473,923–71,475,318 bp (Table 2).

Two sequences that shared 116 bp and 115 bp (both with 100% sequence identity) with *884_200* were found through BLAST searching of Gramene database. These sequences are located on chromosome 6 at positions 42,646,793–42,646,908 bp and 42,646,902–42,647,016 bp, respectively, and the two sequences together cover the whole sequence of the marker *884_200*. These sequences have an overlapping sequence with a gene, *Sb06g015330*, that codes for putative uncharacterized protein Sb06g015330 (source: UniProtKB/TrEMBL; Acc: C5YFS2_SORBI; cited at http://www.gramene.org). The corresponding position of marker *884_200* on sorghum chromosome 6 (GenBank accession number *NC_012875.2*) is 42,689,435–42,689,555 bp (Table 2). Similarly, two sequences that shared 155 bp and 72 bp (with 92.9 and 98.6% sequence identity, respectively) with *835_200* were found through BLAST searching of Gramene database. These sequences are located on chromosome 10 at position 56,232,617–56,232,771 bp and 56,232,771–56,232,842 bp, respectively. The corresponding position of marker *835_200* on sorghum chromosome 10 (GenBank accession number *NC_012879.2*) is 56,491,522–56,491,702 bp (Table 2). Hence, markers *811_1400*, *884_200* and *835_200* are located on chromosomes 3, 6 and 10, respectively. Of these markers, *811_1400* and *884_200* were converted into SCAR markers *SCAR_811* and *SCAR_884*, respectively.

**Fig. 3:**
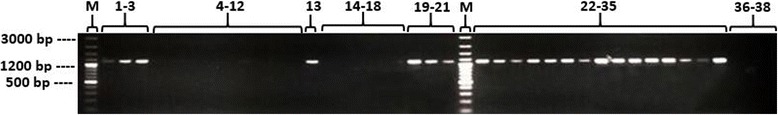
Segregation of *SCAR_811* marker in aluminium tolerant (lanes 4–18) and sensitive (lanes 22–35) F_2:3_ progeny. The F_1_s are on lanes 19–21 and the parental lines *Seredo* and *ICSR 110* are on lanes 1–3 and 36–38, respectively

The first attempt to develop SCAR marker based on the sequence of *811_1400* fragment was by using forward and reverse primers that were developed through extending the sequence of *ISSR_811* primer. These primers amplified fragments of the same size in both parents and their progeny. DNA sequencing of the fragments from tolerant and sensitive parents revealed two SNP sites that differentiate the parents (Table 3). One of these SNPs was targeted to design the forward primer in order to get amplified product specific to the sensitive parent, *Seredo*. The use of a forward primer that included the SNP site and a reverse primer that was designed by extending the *ISSR_811* primer resulted in the development of a polymorphic marker, *SCAR_811*. Fig. 3 shows the *SCAR_811* marker segregation in the F_2:3_ progeny and parents. The Chi-square goodness-of-fit test showed Mendelian segregation of this marker among the F_2:3_ progeny (χ^2^ = 0.14; *P* = 0.71; Table 2). The sensitive parental line and most of the sensitive F_2:3_ progeny bear *SCAR_811* whereas the marker is absent in the tolerant parent *ICSR 110* and most of the tolerant progeny showing the association between the *SCAR_811* marker and tolerance to Al toxicity. This marker explained 10% of the variation in NRL_Al_ (*R*^2^ = 0.10; *P* < 0.001) and 4% of %RRG (*R*^2^ = 0.04; *P* < 0.01) in the F_2:3_ progeny (Table 2).

**Table 3:** Partial DNA sequences of *ICSR 110* and *Seredo* parental lines sequenced by primers designed based on the DNA sequence of *811_1400* marker that was amplified in *Seredo* but not in *ICSR 110*

PL	DNA sequence																																																						
1^a^	**A**	A	A	A	T	A	G	C	A	G	T	A	T	T	T	A	A	G	G	A	C	G	G	A	G	G	G	A	G	T	A	G	C	T	A	T	T	G	A	C	C	G	G	A	T	G	G	A	T	T	C	A	**G**	A	C
2^b^	**G**	A	A	A	T	A	G	C	A	G	T	A	T	T	T	A	A	G	G	A	C	G	G	A	G	G	G	A	G	T	A	G	C	T	A	T	T	G	A	C	C	G	G	A	T	G	G	A	T	T	C	A	**A**	A	C

*PL* parental line, *1*^*a*^ *ICSR 110*; *2*^*b*^ *Seredo*

There are two variable sites that differentiated the two parents as indicated in bold

The primers for *SCAR_884* were developed by excluding the first three nucleotides at the 5′-end of *ISSR_884* primer and extending the sequence at the 3′-end. The *884_200* fragment was specific to *ICSR 110* parental line. However, the newly designed *SCAR_884* primer-pairs amplified fragments both in *Seredo* and *ICSR 110* but of different fragment sizes. Sequencing of these fragments revealed that the fragment from *Seredo* had a 220 bp insertion. All F_1_ individuals produced from the *Seredo x ICSR 110* crosses had both fragments and hence the *SCAR_884* marker is co-dominant and can be used to differentiate homozygotes and heterozygotes at this locus. This marker showed significant association with Al tolerance (*P* < 0.05) when analyzed using both NRL_Al_ (*R*^2^ = 0.017) and %RRG (*R*^2^ = 0.025) indices.

Analysis of the parental lines and some of their F_2:3_ progeny using STS markers *M181* and *CTG29* produced fragments of the same size for each marker, and could not be used to differentiate the tolerant and sensitive lines. Hence, the PCR products of the parental lines as well as five most tolerant and four most sensitive F_2:3_ progeny were sequenced for both markers in an attempt to identify useful sequence variation. The DNA sequences of marker *M181* were identical for all genotypes sequenced whereas a two base pair *indel* (GA/−) that differentiated the sensitive and tolerant parents was observed in the case of *CTG29* (Table 4). Based on these sequences, a new dominant marker, designated as *CTG29_3b* (Table 2), was developed by designing a new forward primer that included the *indel* sequence. The *CTG29_3b* marker amplified by this forward primer and the *CTG29* reverse primer was present in *ICSR 110* and most tolerant F_2:3_ progeny and absent in *Seredo* and most sensitive F_2:3_ progeny. The association of this marker with Al tolerance when analyzed based on NRL_Al_ (*R*^2^ = 0.048) and %RRG (*R*^2^ = 0.040) is significant (*P* < 0.005) (Table 2).

**Table 4:** Partial DNA sequence of STS locus *CTG29* showing indels that differentiated the aluminium tolerant F_2:3_ progenies (15r, 15A2, 21a, 15 m, 21 h, 15v and 42c) and parental line *ICSR 110* from aluminium sensitive F_2:3_ progeny (60f, 59a, 689, 693) and parental line *Seredo*

Sample code	DNA sequence																																					
15r	A	T	A	T	T	A	T	T	A	A	A	A	C	T	G	T	G	T	G	A	T	A	T	A	G	C	G	T	G	A	G	C	G	T	G	G	A	C
15A2	A	T	A	T	T	A	T	T	A	A	A	A	C	T	G	T	G	T	G	A	T	A	T	A	G	C	G	T	G	A	G	C	G	T	G	G	A	C
21a	A	T	A	T	T	A	T	T	A	A	A	A	C	T	G	T	G	T	G	A	T	A	T	A	G	C	G	T	G	A	G	C	G	T	G	G	A	C
15 m	A	T	A	T	T	A	T	T	A	A	A	A	C	T	G	T	G	T	G	A	T	A	T	A	G	C	G	T	G	A	G	C	G	T	G	G	A	C
21 h	A	T	A	T	T	A	T	T	A	A	A	A	C	T	G	T	G	T	G	A	T	A	T	A	G	C	G	T	G	A	G	C	G	T	G	G	A	C
15v	A	T	A	T	T	A	T	T	A	A	A	A	C	T	G	T	G	T	G	A	T	A	T	A	G	C	G	T	G	A	G	C	G	T	G	G	A	C
42c	A	T	A	T	T	A	T	T	A	A	A	A	C	T	G	T	G	T	G	A	T	A	T	A	G	C	G	T	G	A	G	C	G	T	G	G	A	C
ICSR-110	A	T	A	T	T	A	T	T	A	A	A	A	C	T	G	T	G	T	G	A	T	A	T	A	G	C	G	T	G	A	G	C	G	T	G	G	A	C
Seredo	A	T	A	T	T	A	T	T	A	A	A	A	C	T	G	T	G	T	–	–	T	A	T	A	G	C	G	T	G	A	G	C	G	T	G	G	A	C
60f	A	T	A	T	T	A	T	T	A	A	A	A	C	T	G	T	G	T	–	–	T	A	T	A	G	C	G	T	G	A	G	C	G	T	G	G	A	C
59a	A	T	A	T	T	A	T	T	A	A	A	A	C	T	G	T	G	T	–	–	T	A	T	A	G	C	G	T	G	A	G	C	G	T	G	G	A	C
689	A	T	A	T	T	A	T	T	A	A	A	A	C	T	G	T	G	T	–	–	T	A	T	A	G	C	G	T	G	A	G	C	G	T	G	G	A	C
693	A	T	A	T	T	A	T	T	A	A	A	A	C	T	G	T	G	T	–	–	T	A	T	A	G	C	G	T	G	A	G	C	G	T	G	G	A	C

**Fig. 4:**
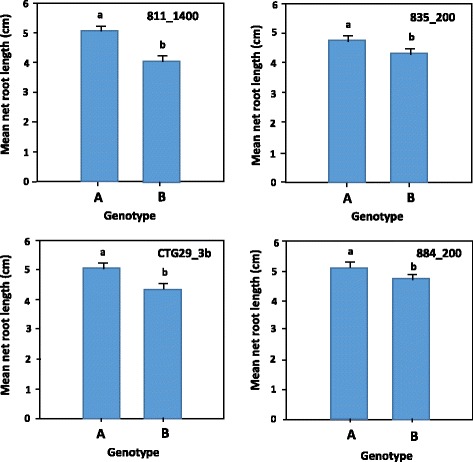
Net root length of sorghum F_2:3_ progeny grown in the presence of 148 μM aluminium based on the different genotypic classes for four dominant markers (*811_1400*, *835_200*, *CTG29_3b* and *884_200*). For each locus, genotypes with different letters indicated on top of the bars were significantly different from each other in their tolerance to Al toxicity. The error bars represent standard error values

The BLAST searching of *CTG29-3b* sequence against the sorghum genome sequence at http://www.gramene.org identified a sequence that share 177 bp (with 100% sequence similarity) with *CTG29-3b* at position 70,979,101–70,979,277 bp on chromosome 3, in agreement with previous reports [23, 24]. The corresponding position of this marker on sorghum chromosome 3 (GenBank accession number *NC_012872.2*) is 70,939,651–70,939,834 bp (Table 2). Overall, single marker analysis revealed that the mean net root length of the different genotypic groups for each of the four dominant markers (*811_1400*, *835_200*, *884_200*, *CTG29_3b*) was significantly different (Fig. 4).

**Fig. 5:**
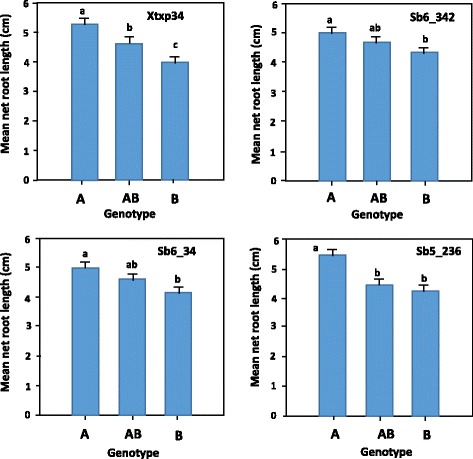
Net root length of sorghum F_2:3_ progeny grown in the presence of 148 μM aluminium based on the different genotypic classes of four codominant markers (*Xtxp34*, *Sb6_342*, *Sb6_34* and *Sb5_236*). For each locus, genotypes that share the same letter indicated on top of the bars were not significantly different from each other in their tolerance to Al toxicity. The error bars represent standard error values

**Fig. 6:**
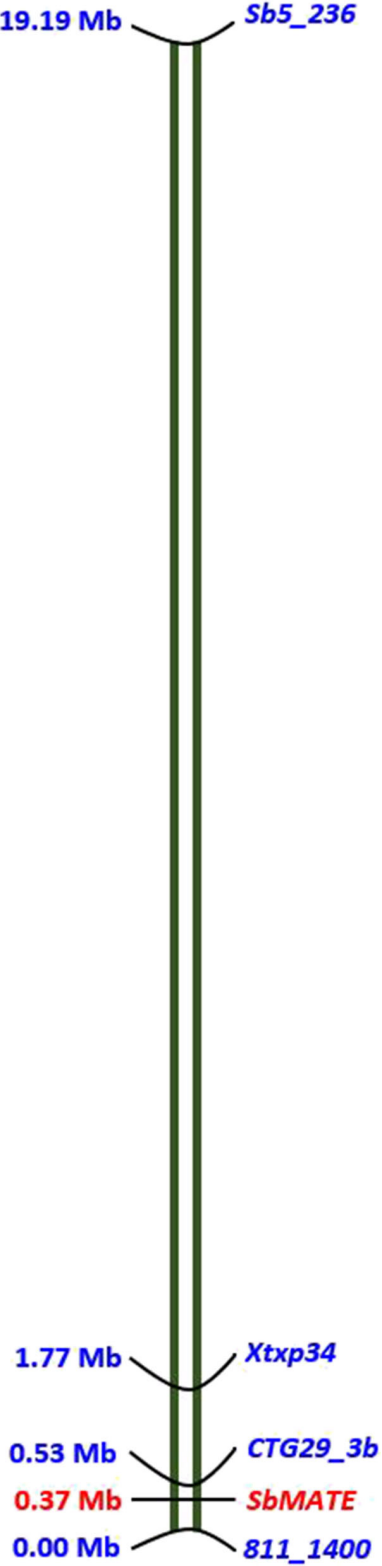
Diagrammatic representation of the four markers on chromosome 3 (*Sb5_236*, *Xtxp34*, *CTG29_3b and 811_1400*) showing their order and distance between them in Mbp

In the case of SSR, polymorphism was detected in nine out of the 24 loci analyzed for the parents. Data analysis based on the 229 F_2:3_ progeny together with the parental lines revealed that markers from four of the nine loci (*Xtxp34*, *Sb6_34*, *Sb5_236* and *Sb6_342*) showed significant association with both NRL_Al_ and %RRG (Table 2; Fig. 5). The mean net root growth of the different genotypic classes, especially the homozygous classes were significantly different (*P* ≤ 0.05; Fig. 5). For marker *Xtxp34*, the mean net root lengths of the three genotypic classes were distinctly different (Fig. 5). For markers *Sb6_342* and *Sb6_34*, the heterozygous classes (AB) had mean net root lengths that were intermediate between the homozygous classes and were not significantly different from both homozygous parents (*P* > 0.05). Whereas the heterozygous class in marker *Sb5_236* had mean net root length that was similar to net root length of the B homozygous class. The NRL_Al_ phenotypic variance (*R*^2^ values) explained by these SSR markers were 14% for *Xtxp34*, 6% for *Sb6_34*, 11% for *Sb5_236* and 4% *Sb6_342*. For %RRG, these values are 7, 2, 5 and 3%, respectively (Table 2). The four markers on chromosome 3 (*811_1400*, *CTG29_3b*, *Xtxp34* and *Sb5_236*) together explained 22% of the variation in NRL_Al_ and 12% of the variation in %RRG (*P* < 0.001). The order of these markers on chromosome 3 (accession number *NC_012872.2*) is *Sb5_236*, *Xtxp34*, *CTG29_3b* and *811_1400* with 17,426 kbp, 1235 kbp and 532 kbp between them in that order (Fig. 6). The closest marker to the *SbMATE* gene is *CTG29_3b* with 166 kbp between them. Overall, the eight markers explained 25% of the variation in NRL_Al_ and 12% of the variation in %RRG (*P* < 0.001).

## Discussion

Several molecular markers that are linked to aluminium tolerance genes have been identified in different crops. In rye, three AFLP markers [36] that are linked to gene *Alt3* and two RAPD markers that were converted to SCAR markers [37], flank *Alt1* gene. In maize, RFLP and SSR markers linked to Al tolerance QTLs have been reported [38]. Raman et al. [39] identified AFLP and microsatellite markers linked to the aluminium tolerance gene located on chromosome 4H in barley. The identification of molecular markers linked to Al tolerance in sorghum through genetic linkage, QTL and association mapping can accelerate the development of high yielding varieties that can withstand Al stress [14, 23–27]. Three ISSR (*811_1400*, *835_200* and *884_200*), one STS (*CTG29_3b*) and four SSR (*Xtxp34*, *Sb6_34*, *Sb5_236* and *Sb6_342*) markers showed significant association with aluminium tolerance in this study. Four of these markers (*811_1400*, *CTG29_3b, Xtxp34* and *Sb5_236*) are located on chromosome 3 whereas markers *884_200*, *Sb6_342, Sb6_34* and *835_200* are located on chromosomes 6, 7, 8 and 10, respectively.

Genes that regulate Al tolerance in plants are shown to have different mode of actions. In rice, two genes that were referred to as sensitive to Al rhizotoxicity1 and 2 (*STAR1* and *STAR2*) were reported to function as an ATP binding cassette (ABC) transporter and have significant contribution in Al tolerance [40]. Yamaji et al. [41] reported a C2H2-type zinc finger transcription factor, Al resistance transcription factor 1 (*ART1*), which regulates the expression of genes related to Al tolerance in rice, including *STAR1* and *STAR2*. In line with these studies, the present study suggests the involvement of several genes in regulating Al tolerance in sorghum, as the markers that showed significant association with Al tolerance are located on five different chromosomes.

To date, only one Al tolerance locus, *Alt*_*SB*_, which underlies the *SbMATE* gene in sorghum, has been identified and mapped to the terminal region of chromosome 3 and characterized in detail [14, 24, 27]. The *SbMATE* gene, codes for aluminium-activated citrate transporter that is highly expressed in root apices of aluminium-tolerant sorghum lines [14]. Caniato et al. [27] developed functional markers within the *SbMATE* gene that can be used for marker assisted selection for aluminium tolerance. In this study, two STS markers *CTG29* and *M181* that are located at 0.2 cM and 0 cM from *Alt*_*SB*_ [14] were tested on Al tolerant and Al sensitive parental lines. STS *M181* was monomorphic and could not be used in the genotyping of the *Seredo* x *ICSR 110* F_2:3_ progeny. The *CTG29_3b* dominant marker that was successfully developed using the nucleotide polymorphic site, showed significant association with Al tolerance and therefore suggests the role of *Alt*_*SB*_ in contributing to Al tolerance in the population studied.

SSRs can be used in cultivar genotyping, genotype identification, genetic diversity assessment and marker-assisted breeding [27, 42]. Matos et al. [43] developed EST-SSRs that are associated with aluminium tolerance in rye. The SSR markers that showed significant association with Al tolerance in the present study are located on chromosomes 3, 7 and 8. SSR marker *Xtxp34* has been mapped to chromosome 3 [41, 44]. *Sb6_342* was mapped to chromosome 7 [44] whereas *Sb5_236* was mapped to chromosome 3 [41, 44], both of which were confirmed in the present study through BLAST searching of the sorghum genome. Similarly, the SSR marker *Sb6_34* was located on chromosome 8 through BLAST search. These results indicate that in addition to *SbMATE*, other genes are involved in conferring Al tolerance in sorghum. Hence, there is a need to build on these findings by screening more markers close to the identified SSRs to ascertain the presence of new Al tolerance genes in these regions.

The *ISSR_811* primer amplified a distinct locus of about1400 bp (*811_1400*) that differentiated the two parents and also showed a significant association with aluminium tolerance in the F_2:3_ population studied. This marker is located close to *SbMATE* gene on chromosome 3 with about 366 kbp between them (Fig. 6), and hence the phenotypic variance of the aluminium tolerance indices explained by this marker is most likely due to *SbMATE* gene. However, *811_1400* (*SCAR_811*) is also located very close to Calmodulin-Binding Transcription Activator 4 (*CAMTA4*) on this chromosome with only 7.3 kbp between them. *CAMTA2* is an activator of Al inducible *ALMT1* expression in *Arabidopsis AtALMT1* [45]. Marker *CTG29-3b* is located closer to *SbMATE* gene (with only 166 kbp between them) than marker *811_1400*. However, *811_1400* explained more phenotypic variance (*R*^2^ = 0.10) than *CTG29_3b* (*R*^2^ = 0.04) in NRL*Al*, in this study. This may suggest the role of *CAMTA4* in regulating *SbMATE* gene in sorghum.

The other two markers on chromosome 3 are located relatively far from *SbMATE* with 17.4 Mbp (*Xtxp34*) and 1.2 Mbp (*Sb5_236*) when compared with the locations of *CTG29_3b* and *811_1400*. However, these markers explained higher phenotypic variance of the aluminium tolerance indices than *CTG29_3b* and *811_1400*, which may suggest the presence of other genes around the vicinity of these markers that influence Al tolerance in sorghum. It is interesting to note that any pair of these markers on chromosome 3 explained more phenotypic variance than the variance they explained separately (data not shown) suggesting the independence of these markers in their contribution to Al tolerance. The four markers together explained 22% of the variance in NRL_Al_ and 12% in %RRG (Table 2), which is higher than the variance explained by any three or two or one of these markers. Hence, combined use of these markers together with markers on other chromosomes is a preferable approach in screening sorghum genetic resources for tolerance to Al toxicity.

The homeologous relationships between genomes of various cereals have been established [39, 46–50]. Previous studies [46–49] have shown that the long arm of wheat chromosome 4D (4DL) is partially homologous to the proximal portion of the short arm of rye chromosome 7R. Moreover, barley chromosome arm 4HL is homologous to the wheat chromosome arm 4DL [39] and a consensus grass comparative map has shown that rice chromosome 3 is homologous to wheat chromosome four [50]. An Aluminium-activated malate transporter 1-like (*ALMT1*-like) gene (LOC110436609) is located at position 43,835,494–43,840,158 bp on sorghum chromosome 6 (accession number *Nc_012875.2*). Marker *884_200* (*SCAR_884*) that showed significant association with aluminium tolerance in this study is located at position 42,689,435–42,689,555 bp on this chromosome. Since this marker is located close to *ALMT1*-like gene with only about 1.1 Mbp between them, and a strong positive correlation between Al induced malate exudation and relative net root growth has already been shown in sorghum [51], the significant association of this marker with aluminium tolerance is likely due to the role of *ALMT*1-like gene in Al tolerance. This also suggests that the sorghum *ALMT1*-like gene is an orthologue of *ALMTI*, as *ALMTI* gene that encodes Al activated malate transporter is known to regulate aluminium tolerance in wheat, barley and rye [17, 52, 53]. Hence, it is important that this gene is investigated in detail in relation to aluminium tolerance in sorghum.

## Conclusion

The four markers on chromosome 3 (*811_1400* or *SCAR_811*, *CTG29_3b*, *Xtxp34* and *Sb5_236*) suggest the role of *Alt*_*SB*_ locus (*SbMATE* gene) in Al tolerance in the sorghum population used in the present study. However, their positions on chromosome 3 in relation to the position of *SbMATE* and the phenotypic variance explained by each of these markers suggest the presence of other genes on this chromosome that play a role in aluminium tolerance in sorghum. The presence of four other markers on different chromosomes that showed significant association with aluminium tolerance suggests the presence of additional genes that contribute to this trait in sorghum. Since the eight markers together explained higher phenotypic variance of the two Al tolerance indices than what explained by individual markers or any other combination of markers, the use of all markers in screening sorghum germplasm for Al tolerance is recommended.
